# A^2^DS^2^ Score Combined With Clinical and Neuroimaging Factors Better Predicts Stroke-Associated Pneumonia in Hyperacute Cerebral Infarction

**DOI:** 10.3389/fneur.2022.800614

**Published:** 2022-02-04

**Authors:** Yaoyao Yu, Tianyi Xia, Zhouli Tan, Huwei Xia, Shenping He, Han Sun, Xifan Wang, Haolan Song, Weijian Chen

**Affiliations:** ^1^Radiology Imaging Center, The First Affiliated Hospital of Wenzhou Medical University, Wenzhou, China; ^2^Department of Radiology, Zhongda Hospital, Medical School of Southeast University, Nanjing, China

**Keywords:** hyperacute cerebral infarction, stroke-associated pneumonia, functional outcome, cerebral small vessel disease, magnetic resonance imaging

## Abstract

**Objective:**

To investigate the predictors of stroke-associated pneumonia (SAP) and poor functional outcome in patients with hyperacute cerebral infarction (HCI) by combining clinical factors, laboratory tests and neuroimaging features.

**Methods:**

We included 205 patients with HCI from November 2018 to December 2019. The diagnostic criterion for SAP was occurrence within 7 days of the onset of stroke. Poor outcome was defined as a functional outcome based on a 3-months MRS score >3. The relationship of demographic, laboratory and neuroimaging variables with SAP and poor outcome was investigated using univariate and multivariate analyses.

**Results:**

Fifty seven (27.8%) patients were diagnosed with SAP and 40 (19.5%) developed poor outcomes. A^2^DS^2^ score (OR = 1.284; 95% CI: 1.048–1.574; *P* = 0.016), previous stroke (OR = 2.630; 95% CI: 1.122–6.163; *P* = 0.026), consciousness (OR = 2.945; 95% CI: 1.514–5.729; *P* < 0.001), brain atrophy (OR = 1.427; 95% CI: 1.040–1.959; *P* = 0.028), and core infarct volume (OR = 1.715; 95% CI: 1.163–2.528; *P* = 0.006) were independently associated with the occurrence of SAP. Therefore, we combined these variables into a new SAP prediction model with the C-statistic of 0.84 (95% CI: 0.78–0.90). Fasting plasma glucose (OR = 1.404; 95% CI: 1.202–1.640; *P* < 0.001), NIHSS score (OR = 1.088; 95% CI: 1.010–1.172; *P* = 0.026), previous stroke (OR = 4.333; 95% CI: 1.645–11.418; *P* = 0.003), SAP (OR = 3.420; 95% CI: 1.332–8.787; *P* = 0.011), basal ganglia-dilated perivascular spaces (BG-dPVS) (OR = 2.124; 95% CI: 1.313–3.436; *P* = 0.002), and core infarct volume (OR = 1.680; 95% CI: 1.166–2.420; *P* = 0.005) were independently associated with poor outcome. The C-statistic of the outcome model was 0.87 (95% CI: 0.81–0.94). Furthermore, the SAP model significantly improved discrimination and net benefit more than the A^2^DS^2^ scale, with a C-statistic of 0.76 (95% CI: 0.69–0.83).

**Conclusions:**

After the addition of neuroimaging features, the models exhibit good differentiation and calibration for the prediction of the occurrence of SAP and the development of poor outcomes in HCI patients. The SAP model could better predict the SAP, representing a helpful and valid tool to obtain a net benefit compared with the A^2^DS^2^ scale.

## Introduction

Acute ischemic stroke is the most common type of stroke, accounting for 80% of all stroke patients (1–3). Stroke-associated pneumonia (SAP) is one of the most common complications of acute ischemic stroke, with an incidence of 6.7–36.98% (4–6). The development of SAP may be due to persistent aspiration and stroke-induced immunodepression (7). Previous studies had shown that SAP always leads to poor functional outcome (8), and increases the hospitalization rate and financial burden (9). Therefore, early identification of SAP high-risk groups and timely treatment are crucial.

Previous studies had identified older age, atrial fibrillation, congestive heart failure, stroke severity, stroke subtype, and dysphagia, as important risk factors (10–13), and established several SAP scales for early prediction, such as the A^2^DS^2^ scale, the ISAN scale and the AIS-APS scale (6, 12, 14). The prediction efficiency of the A^2^DS^2^ scale was better than others (15). However, to date, these risk factors only involve clinical data, and the predictive value of neuroimaging findings, especially from MRI, in SAP remains unclear.

Cerebral small vessel disease (CSVD) is a chronic and cumulative brain disease (16). Neuroimaging findings include lacunes, white matter lesions (WMLs), dilated perivascular spaces (dPVS), microbleeds, and brain atrophy (17). Lacunar infarcts exhibited an independent influence on cognitive function, which is worth noting. More than half of individuals who had their first lacunar stroke and did not have cognitive impairment had mild neuropsychological changes. These small modifications are due to the neuroimaging results of silent multiple lacunar infarctions rather than WMLs (18). CSVD is a chronic and progressive cerebrovascular disease and affects the brain diffusely, so its impact cannot be ignored. CSVD is associated with cognitive impairment, dementia, depression, mobility problems and an increased risk of stroke (19–21), but whether it is related to the occurrence of SAP remains to be verified.

Thus, our study combined imaging findings of CSVD and clinical variables to create a model to predict the occurrence of SAP and poor outcome in hyperacute cerebral infarction (HCI) patients.

## Materials and Methods

### Patient Selection

**Figure 1:**
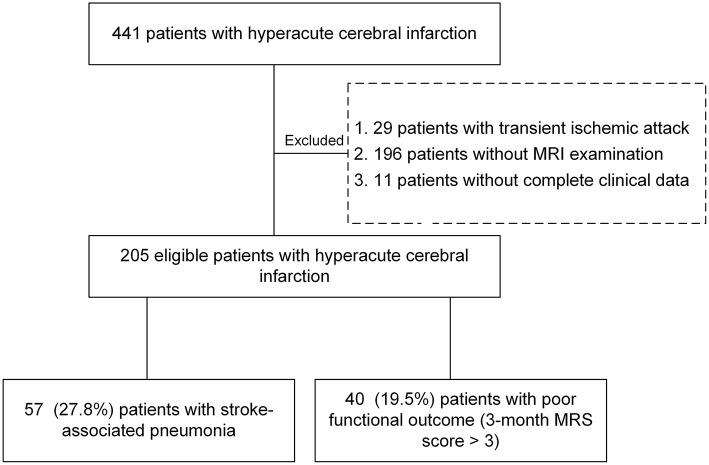
Flow chart for the study patients. A total of 205 patients were included in this study.

In this study, HCI patients with onset >6 h admitted to the Department of Neurology of the First Affiliated Hospital of Wenzhou Medical University from November 2018 to December 2019 were enrolled (2018068). The retrospective study was approved by the Institutional Review Board of our hospital, and the requirement for informed consent was waived. The exclusion criteria were as follows: (1) diagnosed with transient ischemic attack; (2) infection or pyrexia before admission; (3) patients without magnetic resonance imaging (MRI) or with poor quality of MRI; (4) patients who were lost to follow-up or died within 3 months of the ischemic stroke incident; and (5) patients lacking complete clinical data (Figure 1). All patients had obtained a 3-month modified Rankin scale (MRS) score through tele-interview follow-up. Poor outcome was defined as a functional outcome by 3-month MRS score >3, and other patients were divided into a good outcome group.

### Diagnostic Criteria for SAP

SAP was diagnosed by the treating physician according to modified criteria in the United States during the first 7 days after stroke onset (22, 23). Pneumonia was defined as the presence of relevant respiratory clinical symptoms and/or signs, with at least one of the following: white blood cells >11 × 10^9^/L, fever (temperature ≥38.0°C), or a positive chest radiograph, including chest X-ray and computed tomography (24). SAP data were extracted from medical records and reconfirmed through related examinations.

### Image Acquisition

All MR images were performed on a 1.5 T clinical MRI system (MAGNETOM Avanto, SIEMENS, Germany) with an 8-channel head coil. The following MRI sequences were included: T1WI FLAIR [TR/TE = 195/5 ms, inversion time (TI) = 135 ms, slice thickness = 5 mm, slice gap = 6.5 mm, matrix size = 256 × 166, FOV = 187 × 230 mm^2^]; T2WI TSE (TR/TE = 1,000/82 ms, slice thickness = 5 mm, slice gap = 6.5 mm, matrix size = 256 × 192, FOV = 173 × 230 mm^2^); T2WI FLAIR (TR/TE = 9,000/96 ms, TI = 6,237 ms, slice thickness = 5 mm, slice gap = 6.5 mm, matrix size = 256 × 156, FOV = 211 × 260 mm^2^); and DWI (TR/TE = 3,133/89 ms, flip angle = 90°, slice thickness = 5 mm, slice gap = 6.5 mm, matrix = 192 × 192, FOV = 230 × 230 mm^2^, b value =0, 800 s/mm^2^ along three orthogonal directions).

### Clinical, Laboratory and Radiological Data Collection

The basic clinical variables included age, sex, stroke history, hypertension, diabetes, atrial fibrillation, coronary heart disease, smoking, alcohol drinking, and hyperlipidemia. In addition, the patient's condition on admission was recorded, including dysphagia, and blood pressure. Stroke severity and classification were recorded as the National Institute of Health Stroke Scale (NIHSS) score and the TOAST criteria (25). The conscious state of the patients on admission was classified into awake, drowsiness or confusion, and lethargy or coma. According to a previous study, we combined several risk factors to establish the A^2^DS^2^ score (age ≥ 75 years = 1, atrial fibrillation = 1, dysphagia = 2, male = 1, stroke severity: NIHSS score 0–4 = 0, 5–15 = 3, ≥ 16 = 5) (14). Medical treatment of stroke was categorized as intravenous thrombolysis, mechanical thrombectomy, conservative therapy, and others.

Laboratory variables on admission included the absolute count of white blood cells, neutrophils, monocytes, and lymphocytes. The neutrophil-to-lymphocyte ratio (NLR) was the absolute neutrophil count divided by the absolute lymphocyte count (26). Additionally, we collected the absolute count of red blood cells, hemoglobin, fasting plasma glucose, albumin, serum creatinine and estimated glomerular filtration rate. We also collected and measured the radiological variables mainly concentrating on the lesions caused by cerebral infarction and CSVD by MRI (scan within 6 days of admission). Variables related to ischemic stroke localization included cortical involvement and multilobar involvement. The core infarct volume assessment of the increased signal lesion location was completed by 3D reconstruction of the region of interest on axial DWI and continuous multilayer manual depiction of the lesion perimeter (27). We counted the number of lacunes, defined as a round or ovoid subcortical hypoattenuating lesion between 3 and 15 mm in diameter in the territory of one perforating arteriole (17), of cerebrospinal fluid signal intensity on T2 and FLAIR, generally with a hyperintense rim on FLAIR and no increased signal on DWI. Microbleeds were defined as small (<5 mm), homogeneous, round foci of low signal intensity on gradient-echo images (17). A visual rating scale was used to grade the extent of periventricular WMLs (28). Anterior and posterior periventricular WMLs scores were graded from 0 to 2, these scores were summed to yield a total score ranging from 0 to 4. The brain atrophy score was the same as the WMLs score including deep (enlargement of the ventricles) and superficial (enlargement of the sulci) components (graded from 0 to 2, separately), these scores were summed to yield a total score ranging from 0 to 4. The brain atrophy score was determined by comparison with templates indicating normal to atrophied brains in previous research (29). We defined dPVS as small (<3 mm) punctate (if perpendicular) and linear (if longitudinal to the plane of scan) hyperintensities on T2 images in the basal ganglia (BG) or centrum semiovale (CS), and dPVS were graded in a manner similar to that noted for the brain atrophy score and WMLs score (30) (0 = absent dPVS, 1 = <10 dPVS, 2 = 11–20 dPVS, 3 = 21–40 dPVS, 4 = more than 40 dPVS). Neuroimaging variables were evaluated by two trained neuroradiologists (TX and YY, with 4 and 3 years of experience, respectively) blinded to the patients' clinical information and outcome.

### Statistical Analysis

Continuous variables with a normal distribution are presented as the mean ± standard deviation (SD). Non-normal variables are presented as the median [interquartile range (IQR)]. Quantitative variables are presented as numbers (%). The occurrence of SAP and the development of poor outcomes were the outcome variables in our study, so we built SAP and poor outcome models. For categorical variables, differences were calculated using the chi-square test or Fisher's exact test. The student's *t*-test or Mann-Whitney's *U*-test was used to estimate the differences in continuous variables. The logistic regression model was used to analyze the significant variables in univariate analysis (*P* < 0.05) and adopted the forward step-by-step method based on the likelihood ratio. The “rms” software package was used to establish the nomogram based on the multivariate analysis, and Harrell's C-statistics were measured to quantify the discrimination performance of the histogram. The radiomics nomogram was subjected to bootstrapping validation (1,000 bootstrap resamples) to calculate a relatively corrected C-index. Calibration curves were plotted to assess the calibration of the models, along with the Hosmer-Lemeshow test. The forest map showing the results of the multivariate analysis was generated using the “forest” software package. Two-tailed *P* < 0.05 was considered to be statistically significant. The Delong test was used to compare the C-statistics with statistical significance, and decision curve analysis (“rmda” software package) was used to evaluate the prediction net benefit of the A^2^DS^2^ scale and the SAP model for the prediction of SAP. The statistical analysis results were analyzed using SPSS (version 24.0) and R software (version 4.0.3).

## Results

### Univariable Analysis for the Occurrence of SAP

**Figure 2:**
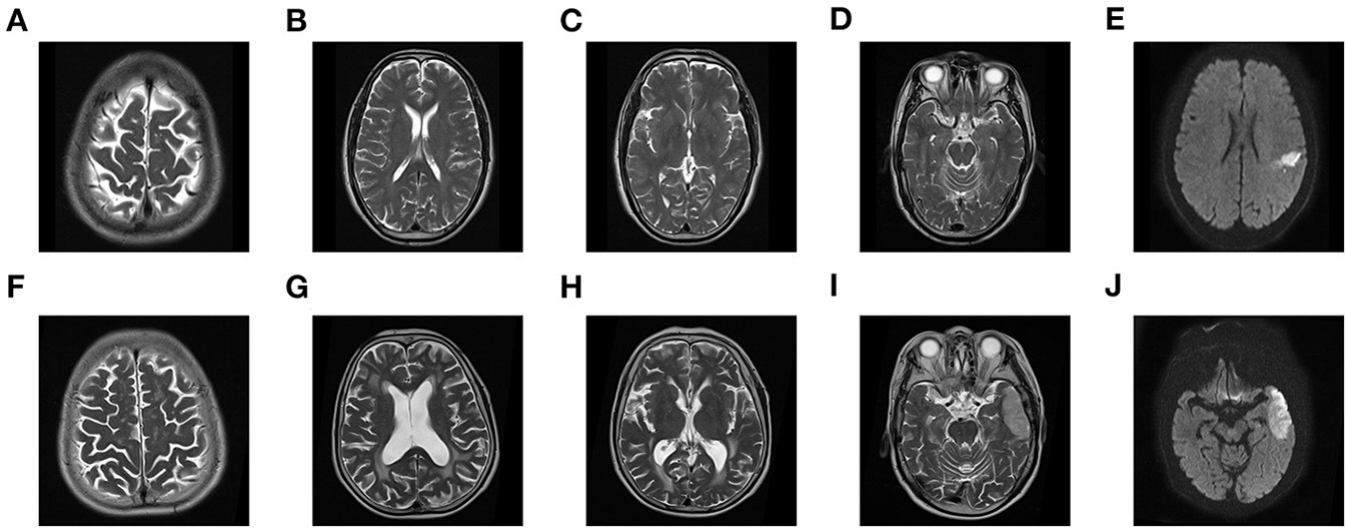
MR features of brain atrophy and core infarct volume. **(A)** 68-year-old patient developed non-SAP and good functional outcome (MRS, 0 points) with brain atrophy 0 level **(A–D)** and core infarct volume 54.6 × 50 ML **(E)**. A 70-year-old patient developed SAP and poor functional outcome (MRS, 5 points) with brain atrophy 2 level **(F–I)** and core infarct volume 419 × 50 ML **(J)**.

A total of 205 patients met all eligibility criteria and were retained for analyses. 57 (27.8%) patients were diagnosed with SAP, and 40 (19.5%) developed poor outcomes. The variables of the patients were shown in Table 1. During their admission, three individuals died of neurological events, two of whom acquired SAP, accounting for 3.5 per cent of SAP patients. The group with SAP had a higher NIHSS score and A^2^DS^2^ score and was older than the non-SAP group (all *P* < 0.05). In addition, patients with a previous stroke, coronary artery disease, dysphagia and poor consciousness status had an increased probability of SAP (all *P* < 0.05). There were differences in some neuroimaging findings, such as multiple lobes involved, core infarct volume and brain atrophy score (all *P* < 0.05). The group with SAP had more severe brain atrophy and larger lesions than the non-SAP group (Figure 2). However, no significant difference in the other neuroimaging findings of CSVD were noted between the patients with SAP and the controls. In addition, some characteristics of laboratory examination did not differ significantly (all *P* > 0.05). Higher absolute counts of white blood cells, neutrophils, and monocytes, NLR and fasting plasma glucose levels were significantly more likely in those who went on to develop SAP. No significant difference in the treatment methods were noted between the two groups (*P* = 0.094), except for mechanical ventilation. Among the 205 patients, 17 (11.5%) non-SAP patients had poor outcomes and 23 (40.4%) SAP patients had poor outcomes at 3 months (*P* < 0.001).

**Table 1:** Univariable analysis of baseline characteristics associated with occurrence of SAP and development of poor outcome.

**Variables**	**Non-SAP** **(*n* = 148)**	**SAP** **(*n* = 57)**	***P*** **-value**	**Good outcome (*n* = 165)**	**Poor outcome** **(*n* = 40)**	***P*** **-value**
**Demographics**						
Male sex, *n* (%)	52 (35.1)	16 (28.1)	0.336	54 (32.7)	14 (35.0)	0.784
Age at onset (years), median (IQR)	65 (57–72)	70 (63–79)	0.022	66 (57–73)	70 (66–73)	0.056
**Medical history**						
Alcohol drinking, *n* (%)	54 (36.5)	23 (40.4)	0.609	60 (36.4)	17 (42.5)	0.472
Smoking, *n* (%)	61 (41.2)	23 (40.4)	0.910	68 (41.2)	16 (40.0)	0.889
Hypertension, *n* (%)	101 (68.2)	44 (77.2)	0.207	110 (66.7)	35 (87.5)	0.009
Diabetes, *n* (%)	48 (32.4)	15 (26.3)	0.395	45 (27.3)	18 (45.0)	0.029
Hyperlipidemia, *n* (%)	15 (10.3)	3 (5.4)	0.267	17 (10.5)	1 (2.6)	0.213
History of malignancy, *n* (%)	8 (5.4)	1 (1.8)	0.446	7 (4.2)	2 (5.0)	1.000
Previous stroke, *n* (%)	25 (16.9)	18 (31.6)	0.021	27 (16.4)	16 (40.0)	0.001
Atrial fibrillation, *n* (%)	27 (18.2)	17 (29.8)	0.070	32 (19.4)	12 (30.0)	0.143
Coronary artery disease, *n* (%)	9 (6.1)	9 (15.8)	0.028	14 (8.5)	4 (10.0)	1.000
**Characteristics of condition on admission**						
Dysphagia, *n* (%)	9.0 (6.1)	19 (33.3)	<0.001	14 (8.5)	14 (35.0)	<0.001
Headache, *n* (%)	8.0 (5.4)	3.0 (5.3)	1.000	10 (6.1)	1 (2.5)	0.613
Consciousness, *n* (%)			<0.001			0.001
Awake	138 (93.2)	33 (57.9)		145 (87.9)	26 (65.0)	
Drowsiness or confusion	6 (4.1)	14 (24.6)		11 (6.7)	9 (22.5)	
Lethargy or coma	4 (2.7)	10 (17.5)		9 (5.5)	5 (12.5)	
Systolic BP (mmHg), mean ± SD	155.1 ± 22.4	154.0 ± 23.7	0.769	154.1 ± 22.4	157.4 ± 24.0	0.424
Diastolic BP (mmHg), mean ± SD	87.2 ± 13.9	85.4 ± 14.6	0.404	86.8 ± 14.6	86.2 ± 12.0	0.814
NIHSS score, median (IQR)	3 (1–7)	8 (3–15)	<0.001	3 (1–7)	9 (4–14)	<0.001
A^2^DS^2^ score, median (IQR)	1 (1–4)	5 (3–6)	<0.001	2 (1–4)	4 (3–6)	<0.001
TOAST classification^a^, *n* (%)			0.129			0.580
Large-artery atherosclerosis	120 (81.1)	41 (71.9)		131 (79.4)	30 (75.0)	
Cardioembolism	25 (16.9)	15 (26.3)		31 (18.8)	9 (22.5)	
**Characteristics of MRI**						
Cortical involvement, *n* (%)	66 (44.6)	34 (59.6)	0.053	78 (47.3)	22 (55.0)	0.380
Multiple lobes involved, *n* (%)	61 (41.2)	36 (63.2)	0.005	71 (43.0)	26 (65.0)	0.013
Core infarct volume (50mL), median (IQR)	0.05 (0.01–0.22) (14.0–221.5)	0.20 (0.03–1.74)	0.001	0.06 (0.02–0.30)	0.20 (0.02–1.35)	0.027
**Small vessel diseases**						
Lacunes ≥2, *n* (%)	33 (22.3)	16 (28.1)	0.385	36 (21.8)	13 (32.5)	0.155
Microbleeds, *n* (%)	18 (12.2)	6 (10.5)	0.774	16 (9.7)	8 (20.0)	0.123
Brain atrophy score, median (IQR)	2 (1–4)	3 (2–4)	0.001	3 (1–4)	3 (2–4)	0.030
Cortical atrophy score, median (IQR)	1 (1–2)	2 (1–2)	0.002	1 (1–2)	2 (1–2)	0.088
Deep atrophy score, median (IQR)	1 (0–2)	2 (1–2)	0.002	1 (0–2)	2 (1–2)	0.014
WMLs score, median (IQR)	1 (1–3)	1 (1–2)	0.733	1 (1–3)	2 (1–3)	0.023
Anterior-WMLs score, median (IQR)	1 (1–1)	1 (1–1)	0.153	1 (1–1)	1 (1–2)	0.032
Posterior-WMLs score, median (IQR)	0 (0–1)	0 (0–1)	0.614	0 (0–1)	1 (0–2)	0.057
dPVS score, median (IQR)	3 (2–5)	3 (2–4)	0.210	3 (2–4)	3 (2–5)	0.208
CSO-dPVS score, median (IQR)	1 (1–2)	1 (1–2)	0.152	1 (1–2)	1 (1–3)	0.905
BG-dPVS score, median (IQR)	2 (1–2)	2 (1–2)	0.508	1 (1–2)	2 (1–3)	0.015
**Characteristics of laboratory examination on admission**						
WBC (× 10^9^/L), median (IQR)	6.83 (5.93–8.91)	9.18 (6.48–10.62)	0.001	6.87 (5.96–9.42)	8.52 (6.84–10.31)	0.023
Neutrophil (× 10^9^/L), median (IQR)	4.42 (3.48–6.39)	6.69 (4.63–8.40)	<0.001	4.56 (3.53–6.64)	6.57 (4.55–8.32)	0.002
Monocyte (× 10^9^/L), median (IQR)	0.46 (0.35–0.61)	0.51 (0.38–0.72)	0.035	0.46 (0.36–0.61)	0.57 (0.33–0.72)	0.177
Lymphocyte (× 10^9^/L), median (IQR)	1.64 (1.21–2.20)	1.39 (1.06–1.93)	0.055	1.61 (1.18–2.20)	1.34 (1.10–1.96)	0.168
NLR, median (IQR)	2.62 (1.88–4.63)	4.07 (2.68–7.34)	<0.001	2.69 (1.88–4.79)	4.03 (2.70–7.06)	0.002
RBC (× 10^12^/L), mean ± SD	4.62 ± 0.49	4.48 ± 0.54	0.070	4.58 ± 0.50	4.57 ± 0.54	0.837
Hemoglobin (g/L), mean ± SD	142.2 ± 15.9	137.6 ± 18.2	0.081	140.8 ± 16.5	141.4 ± 17.7	0.832
Fasting plasma glucose (mmol/L), median (IQR)	5.3 (4.7–7.0)	6.1 (4.9–7.5)	0.019	5.3 (4.6–6.7)	7.05 (5.53–10.55)	0.000
Albumin (g/L), median (IQR)	37.4 (35.3–39.3)	36.0 (33.5–39.4)	0.076	37.3 (35.3–39.6)	35.3 (33.8–38.7)	0.050
Scr (μmol/L), median (IQR)	70 (59–82)	74 (57–85)	0.499	68 (59–82)	74 (65–84)	0.197
eGFR (mL/min/1.73m^2^), median (IQR)	94 (81–104)	86 (73–102)	0.162	94 (79–106)	87 (72–100)	0.152
**Clinical therapy**						
Mechanical ventilation, *n* (%)	1 (0.7)	4 (7.0)	0.033	2 (1.2)	3 (7.5)	0.082
Treatment methods, *n* (%)			0.094			0.161
Intravenous thrombolysis	46 (31.1)	17 (29.8)		51 (30.9)	12 (30.0)	
Mechanical thrombectomy	5 (3.4)	6 (10.5)		6 (3.6)	5 (12.5)	
Conservative therapy	89 (60.1)	28 (49.1)		96 (58.2)	21 (52.5)	
Others^b^	8 (5.4)	6 (10.5)		12 (7.3)	2 (5.0)	
Occurrence of SAP, *n* (%)				34 (20.6)	23 (57.5)	<0.001
MRS score at 3 months, median (IQR)	1 (0–1)	2 (0–5)	<0.001			
Poor outcome group at 3 months (MRS Score >3), *n* (%)	17 (11.5)	23 (40.4)	<0.001			

*Continuous variables with normal distribution were presented as mean ± standard deviation (SD); non-normal variables were presented as median [interquartile range (IQR)]; quantitative variables were presented as number (%). NIHSS, National Institute of Health stroke scale; WMLs, white matter lucencies; dPVS, dilated perivascular space; CSO, centrum semiovale; BG, basal ganglia; WBC, white blood cell; NLR, neutrophil-lymphocyte ratio; RBC, red blood cell; Scr, serum creatinine; eGFR, estimated glomerular filtration rate; SAP, Stroke-associated pneumonia; MRS, modified Rankin scale*.

a *Some patients were excluded from this group, due to the sample size of patients classified by other TOAST classifications was too small to perform chi-square tests*.

b *Other treatment methods included intravenous thrombolysis combined with mechanical thrombectomy, stent implantation and balloon dilation*.

### Univariable Analysis for the Development of Poor Outcomes

**Figure 3:**
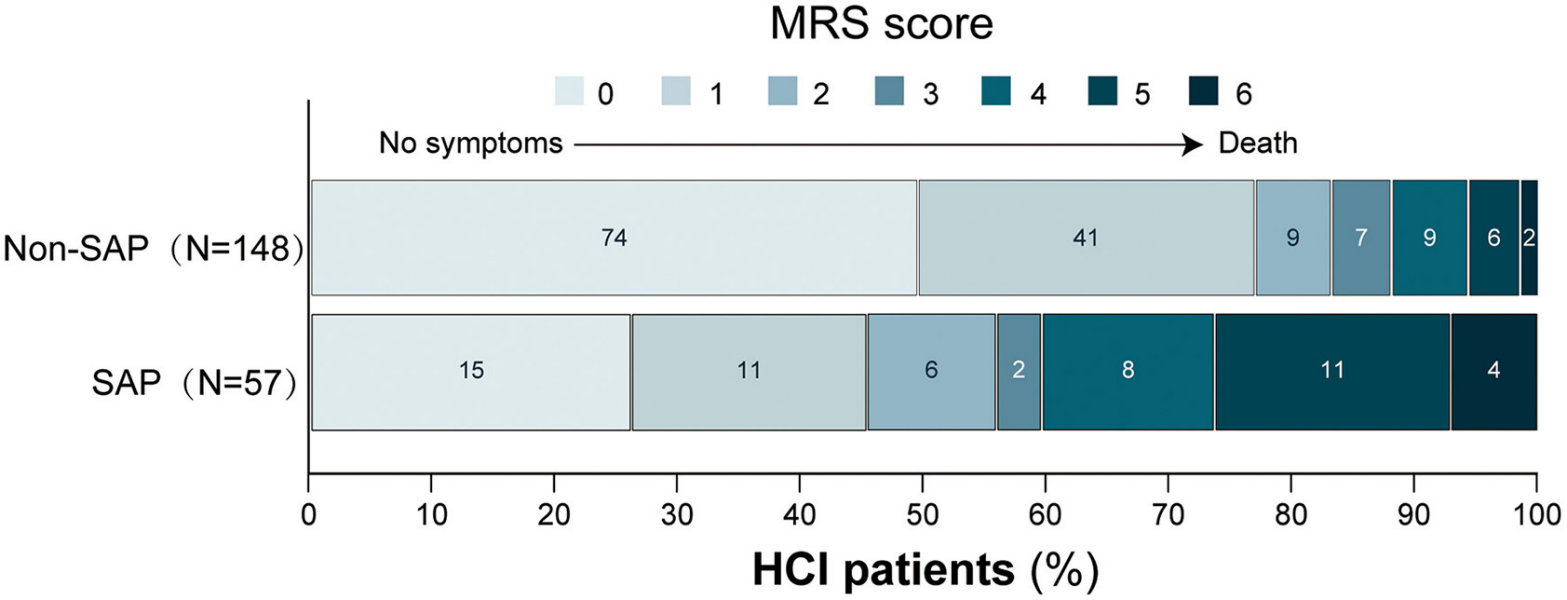
Distribution of MRS scores at 3 months, according to with/without SAP. Scores on MRS range from 0 to 6. The numbers in the cell represent frequencies. The y-axis measures the cumulative proportion of MRS scores. MRS, modified Rankin scale; SAP, stroke-associated pneumonia.

Patients with poor outcomes more frequently had a history of hypertension, diabetes, previous stroke, and dysphagia, and a higher NIHSS score and were more likely to have poor consciousness status on admission (all *P* < 0.05). Poor outcome patients had a larger core infarct volume, higher brain atrophy score, WMLs score and BG-dPVS score, and more frequently had multiple lobes involved (all *P* < 0.05). Furthermore, higher absolute count of white blood cells and neutrophils, and NLR, fasting plasma glucose levels, and lower albumin levels were significantly more likely in those who went on to develop a poor outcome (all *P* < 0.05). Similar to SAP results, no significant difference in the treatment methods were noted between the poor outcome group and the good outcome group (*P* = 0.162). Patients with poor outcomes had a more frequent occurrence of SAP (*P* < 0.001). The detailed distribution of functional outcomes at 3 months in patients with and without SAP was shown in Figure 3.

### Independent Factors and Development of Prediction Models

**Figure 4:**
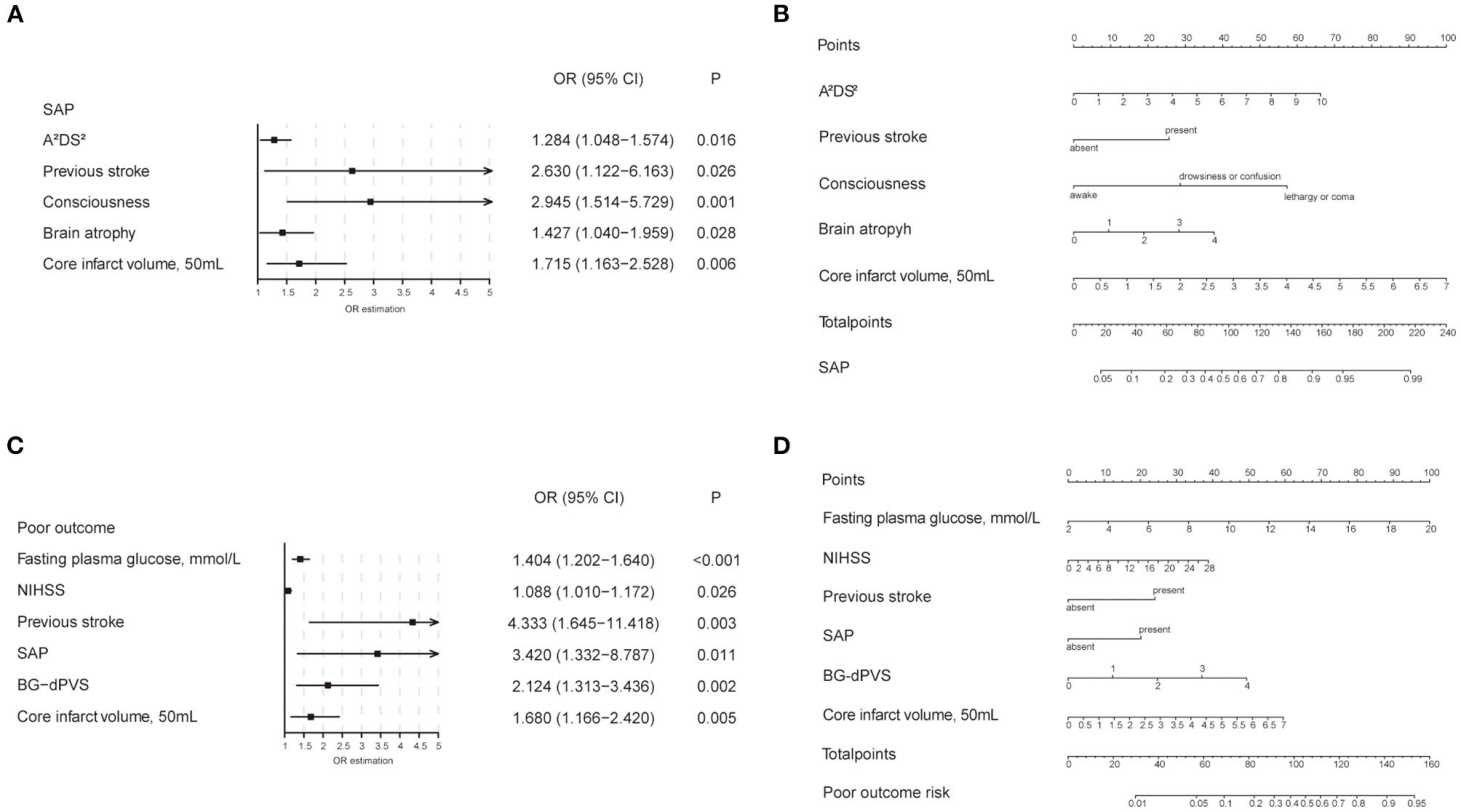
Forest plot and nomogram of independent predictors of SAP and poor outcome. **(A)** Forest plot of independent predictors of SAP with a multivariate regression model. **(B)** The SAP model presented with a nomogram scaled by the proportional regression coefficient of each predictor. **(C)** Forest plot of independent predictors of poor outcome with a multivariate regression model. **(D)** The poor outcome model presented with a nomogram scaled by the proportional regression coefficient of each predictor. SAP, stroke-associated pneumonia; NIHSS, National Institute of Health stroke scale; BG-dPVS, basal ganglia dilated perivascular space; OR, odds ratio.

**Figure 5:**
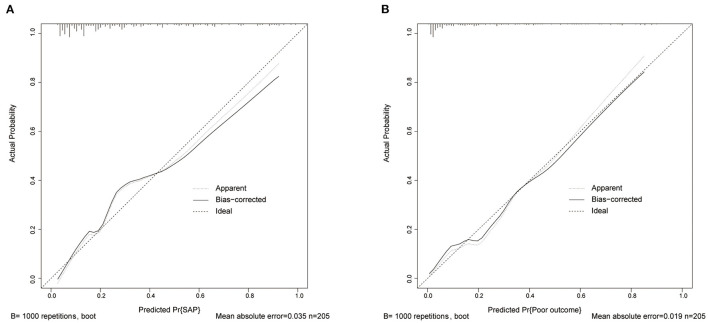
Calibration curve of SAP and poor outcome models in data. **(A)** Calibration curve of the SAP model. **(B)** Calibration curve of the outcome model. SAP, stroke-associated pneumonia.

A logistic regression analysis identified the A^2^DS^2^ score (OR 1.28, 95% CI 1.05–1.57), previous stroke (OR 2.63, 95% CI 1.12–6.16), consciousness (OR 2.945, 95% CI 1.514–5.729), brain atrophy (OR 1.43, 95% CI 1.04–1.96) and core infarct volume (per 50 ml) as independent predictors for the occurrence of SAP (Figure 4A). The SAP model that incorporated the above independent predictors were developed and presented as a nomogram (Figure 4B). Like the previous model, fasting plasma glucose (OR 1.40, 95% CI 1.20–1.64), NIHSS score (OR 1.09, 95% CI 1.01–1.17), SAP (OR 3.42, 95% CI 1.33–8.79), BG-dPVS score (OR 2.12, 95% CI 1.31–3.44) and core infarct volume (per 50 ml) (OR 1.68, 95% CI 1.17–2.42) were independent predictors for the development of poor outcome at 3 months (Figure 4C). The outcome model was developed and presented as the nomogram with the above factors (Figure 4D). The C-statistic of the SAP model was 0.84 (95% CI 0.78–0.90), and that of the outcome model was 0.87 (95% CI 0.81–0.94). The calibration curve of the SAP and outcome models showed good agreement between prediction and actuality, which was confirmed by bootstrapping validation (Figure 5).

### Predictive Value of the A^2^DS^2^ Score and SAP Model

**Figure 6:**
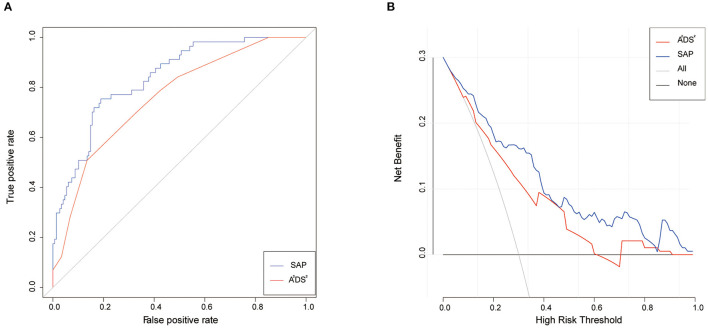
Receiver-operating characteristic analysis and decision curve analysis for the A^2^DS^2^ and SAP model to the prediction of SAP. **(A)** the C-statistics for the A^2^DS^2^ (red line) and SAP model (blue line) in the data. **(B)** The y-axis measures the net benefit. The red line represents the A^2^DS^2^. The blue line represents the SAP model. The gray line represents the assumption that all patients have SAP. The black line represents the assumption that no patients have SAP. SAP, stroke-associated pneumonia.

The A^2^DS^2^ score yielded a C-statistic of 0.76 (95% CI 0.69–0.83) for the prediction of the occurrence of SAP. However, the C-statistic of the SAP model was 0.84 (95% CI 0.78–0.90), which was much higher than the A^2^DS^2^ scale (Figure 6A). The chi-square test yielded a significant difference between the A^2^DS^2^ score and the SAP model (*P* < 0.001). The decision curve analysis for the SAP model and A^2^DS^2^ score is presented in Figure 6B. The decision curve showed that using the SAP model to predict the occurrence of SAP adds more benefit than either the treat-all-patients scheme or the treat-none scheme, compared with the A^2^DS^2^ score.

## Discussion

At present, SAP often brings significant physical harm to patients, and affects the prognosis and survival rate of patients with HCI. For long-term consideration, it is necessary to assess the possibility of the occurrence of SAP to intervene early. To our knowledge, we were the first study to explore the relationship between CSVD and SAP. We found that brain atrophy was significantly associated with SAP, and that dPVS was independently associated with poor outcomes. In addition, we showed that neuroimaging findings combined with A^2^DS^2^ can effectively predict the occurrence of SAP. CSVD can be precisely examined by MRI to help clinicians identify high-risk patients at the early stages of SAP and reduce the financial and patient care burden.

In our study, SAP occurred in 27.8% of patients with HCI, which is similar to that reported in previous studies (4–6). Similarly, our study showed that SAP was an independent risk factor, and the incidence of poor function outcome was 57.5%. In the study of Hong et al. (8) and Finlayson et al. (31), percentages of SAP patients with a poor prognosis were 29.5 and 63.7%, respectively. Patients with a higher A^2^DS^2^ score were more likely to develop SAP, which was consistent with previous studies (31, 32). Li et al. demonstrated that patients with coma and other impaired consciousness statuses at the time of stroke attack had a significantly higher incidence of pneumonia, which was consistent with our study (33). While it is known that disorders of consciousness are correlated to diffuse injury to the cerebral cortex or thalamus or damage to the ascending reticular activating system as a result of brain stem damage, our study found that patients with lesions that did not involve the basal ganglia were more likely to keep awake than others, with incidence rates of 86.9 and 79.6%, respectively. Some studies had shown that dysphagia was related to the location of infarction (34), such as Kim et al. There was no statistically significant difference between the occurrence of dysphagia and the different lesions including lobe, basal ganglia, thalamus, and subtentorial structure in our study. The majority of patients with the anterior circulation involvement in 165 subjects (80.5%) and posterior circulation in 40 subjects (19.5%), so symptoms associated with subtentorial structural damage may not be present. In addition, the previous stroke also correlated with the occurrence of SAP and the development of poor outcomes. Fasting blood glucose levels on admission were linked to functional outcomes in our study, while pre-existing diabetes was not. Hyperglycemia, but not a history of diabetes, was an independent risk for functional outcomes, probably because of the way hyperglycemia impacts inflammation and angiotensin-converting enzyme 2 expression. Patients with higher glucose levels may be more sensitive to redox-mediated reperfusion harm, resulting in poor functional outcomes (35). The NIHSS scale is a simple, effective, repeatable, and safe scale. It is the most commonly used scale for the assessment of neurological impairment during acute stroke (36).

Previous studies derived that WMLs can predict the development of SAP (37), and WMLs were statistically significant in our univariate analysis. However, we found that brain atrophy was independently correlated with the occurrence of SAP. Gray matter atrophy was usually progressive and documented in patients with stroke primarily in the lacunar type, and the previous study had shown that frontal lobe dysfunction and local cortical and subcortical Gray matter atrophy were the best ways to distinguish the clinical course of lacunar infarct patients with or without cognitive impairment (38). CSVD burden was associated with patients' brain atrophy. The specific mechanism by which CSVD induces brain atrophy had remained a mystery. CSVD may cause additional degeneration, such as cortical atrophy caused by WMLs tract disconnection (39–41). Furthermore, some research had discovered a relationship between lacunar infarction and brain atrophy (41, 42). Elderly patients with severe cortical atrophy had a significantly higher risk of lower respiratory tract infections, possibly because severe brain atrophy led to difficulty swallowing and indirectly to the development of pulmonary infections (43). It is worth noting that the occurrence of dPVS in this study was independently associated with poor outcomes in HCI patients, especially the significant correlation between BG-dPVS and 3-month outcome in HCI patients, which had not been previously reported. The association between dPVS and outcome may also be due to impaired inter tissue fluid drainage (and any excess fluid resulting from blood-brain barrier dysfunction) by impairment of perivascular blood flow, impeding the clearance of tissue metabolites, including beta-amyloid and other proteins (19). This biological mechanism had been demonstrated in Alzheimer's disease, brain amyloid vascular disease and some single-gene small vessel diseases (44). In addition to neuroimaging findings of CSVD, we found that infarct volume predicts not the only poor outcome, but also the occurrence of SAP, which was consistent with the findings of another study (45).

Studies have shown that the A^2^DS^2^ score has high clinical applicability for stroke patients, and is an effective scale for the early prediction of the occurrence of SAP and the prognosis of stroke patients (14, 46). However, the efficacy of the A^2^DS^2^ score alone still needed to be improved, and the C-statistic of the A^2^DS^2^ score in our study was 0.76. After we added previous stroke, awareness on admission, core infarct volume and brain atrophy, the C-statistic of the new SAP predictive model increased to 0.84. Our study found that the SAP model provided better differentiation and greater clinical benefits than the A^2^DS^2^ scale. The C-statistic was 0.87 in the outcome model.

There are several important practical implications. It may be helpful for clinicians to identify those at risk of the occurrence of SAP and the development of poor outcomes in HCI patients. Once this high-risk group is identified, early initiation of therapy may be considered to improve the functional outcome of patients. The advantages of our study were as follows. First, it was the first study to explore the association between SAP and CSVD. Second, we also proposed models to predict the occurrence of SAP and the development of poor outcomes, continuing the findings of previous research (13, 14, 46), and improving the ability of clinical prediction. However, some limitations in our study should be noted. First, our study was a retrospective study from a single center, which could result in insufficient samples and may not be applicable to non-Asian populations. Second, this study did not have a validation group, and self-validation by repeated sampling may lead to overfitting of the model. In addition, our study was conducted in patients with HCI and may not be fully applicable to all ischemic stroke patients. Although these findings may be interpreted as underlying risk factors, larger prospective studies may be needed for further validation. These findings and models may be informative for clinicians managing HCI patients and help with long-term prognostication and management decisions.

## Conclusion

In summary, the neuroimaging findings of CSVD combined with the A^2^DS^2^ score can better predict the occurrence of SAP and the development of poor outcomes of patients with HCI. In clinical practice, the addition of MRI examination helps to detect SAP and predict patient prognosis.

## Data Availability Statement

The original contributions presented in the study are included in the article/Supplementary Material, further inquiries can be directed to the corresponding author/s.

## Ethics Statement

This study was approved by the Ethics Committee of the First Affiliated Hospital of Wenzhou Medical University (2018068). The Institutional Review Board of the hospital waived the requirement for informed consent.

## Author Contributions

YY analyzed the data, framed it, and wrote the manuscript. TX analyzed data and edited manuscripts. ZT and HX collected the imaging data. SH, HSu, XW, and HSo collected the clinical data. WC designed and supervised the paper, and finalized the manuscript. All authors reviewed the manuscript and made significant contribution to this manuscript.

## Conflict of Interest

The authors declare that the research was conducted in the absence of any commercial or financial relationships that could be construed as a potential conflict of interest.

## Publisher's Note

All claims expressed in this article are solely those of the authors and do not necessarily represent those of their affiliated organizations, or those of the publisher, the editors and the reviewers. Any product that may be evaluated in this article, or claim that may be made by its manufacturer, is not guaranteed or endorsed by the publisher.
